# Caregiver-reported disease burden in Krabbe disease: evaluating outcomes of hematopoietic stem cell transplantation

**DOI:** 10.1186/s13023-025-04176-3

**Published:** 2026-01-07

**Authors:** Nicholas Alexander Bascou, Skyler Jackson, Patti Engel, Anne Melchior, Paul Orchard, Stacy Pike-Langenfeld

**Affiliations:** 1 Department of Neurology, Johns Hopkins University, 1800 Orleans Street, Baltimor, MD USA; 2 Krabbe Connect, Rosemount, MN USA; 3 Engage Health, Eagan, MN USA; 4 Division of Pediatric Bone Marrow Transplantation, University of Minnesota, Minneapolis, MN USA

**Keywords:** Krabbe disease (KD), Infantile krabbe disease (IKD), Late-infantile krabbe disease (LIKD), Hematopoietic stem cell transplantation (HSCT), Newborn screening (NBS), Caregiver reported outcomes, Patient reported outcomes, Globoid cell leukodystrophy

## Abstract

**Background:**

Krabbe disease (KD) is a rapidly progressive neurodegenerative disorder caused by β-galactocerebrosidase deficiency. While KD has been added to the Recommended Uniform Screening Panel (RUSP), only 15 states have an active KD newborn screening (NBS) program. It is uncertain at what rate states will adopt RUSP recommendations, with a frequently cited barrier being the absence of investigations addressing the impact of hematopoietic stem cell transplantation (HSCT) on quality-of-life.

**Methods:**

We developed a 90-minute caregiver interview to gather qualitative and quantitative data (including the validated Leukodystrophy Quality-of-Life Assessment – LQLA) evaluating patient/family-centered outcomes of HSCT. The interview was designed to explore the following: 1) disease burden on the patient; 2) physical burden on the caregiver; and 3) emotional/social burden on the caregiver. Comparisons were made between children not transplanted/transplanted late and children transplanted early. Infantile KD (IKD) and late infantile KD (LIKD) were analyzed independently.

**Results:**

Forty caregivers participated (non-transplanted/transplanted late: IKD = 19, LIKD = 7; transplanted early: IKD = 10, LIKD = 4). Analysis of the LQLA revealed a relative reduction in disease burden in both IKD and LIKD groups who were transplanted early. Specifically, the early transplanted cohorts achieved statistically significant higher overall scores on the LQLA, as well as better scores in various subcategories in comparison to their non-transplanted/transplanted late counterparts. For IKD, analysis of Likert scale and weighted analysis demonstrated a tendency towards decreased physical burden on caregivers of children transplanted early. Although all groups experienced significant social/emotional burdens, caregivers of IKD transplanted early benefitted from improved sleep, mental health, and familial/spousal relationships compared to IKD non-transplanted/transplanted late.

**Conclusion:**

This study provides convincing evidence that HSCT improves quality-of-life and reduces caregiver burden in IKD. The evidence is somewhat less clear for LIKD due to the small LIKD sample size. This data will be critical in the decision-making process for states not currently screening for KD but debating the addition of KD to their NBS panels. Lastly, it will allow families to weigh the risks and benefits of HSCT more confidently when contemplating the life-altering decision of whether to proceed with transplantation.

**Supplementary Information:**

The online version contains supplementary material available at 10.1186/s13023-025-04176-3.

## Introduction

Krabbe disease (KD) is a neurological disorder caused by deficiency of the lysosomal enzyme β-galactocerebrosidase [1]. With insufficient enzymatic activity, galactocerebroside and psychosine accumulate, leading to demyelination within the central and peripheral nervous system [2]. The disease is genetic and inherited in an autosomal recessive pattern [3]. GALC is the causative gene, with over 200 pathogenic variants identified. [4, 5]. Overall, KD prevalence has been estimated at 1/100,000, making it an ultra-rare disease [6–8].

The severity and age of onset for KD are dependent upon the genotype and corresponding enzyme activity and psychosine level [9–11]. Infantile Krabbe disease (IKD) is estimated to account for approximately 85% of cases [6, 12]. It is the most severe phenotype, with onset of symptoms before 12 months of age and a mean survival of 2 years if left untreated. Signs and symptoms of IKD include irritability, regression of psychomotor development, feeding difficulties, hypertonicity, seizures, loss of vision and hearing, and early death [7, 13–15]. The second most common form of Krabbe disease is late-Infantile Krabbe disease (LIKD), with onset between 1 and 3 years of life. Although LIKD is less severe than IKD, patients are substantially impaired with death typically occurring in childhood [6, 16, 17]. The other forms of Krabbe disease are juvenile and adult, which are not as well-described and are not part of this study [5, 18–22].

Regarding treatment, the only established disease-modifying therapy to date is hematopoietic stem cell transplantation (HSCT), with numerous prospective and retrospective studies from Escolar, Kurtzberg and others providing evidence for favorable outcomes in IKD and LIKD phenotypes [23–29]. Nevertheless, there are no prior studies that have systematically investigated parent/caregiver-reported outcomes of HSCT and related improvements in quality of life. Moreover, with increasing focus on patient experience data when designing clinical trials for potential therapies, there is a critical need to explore patient/family-centered experiences and their daily struggles [30–33].

In light of recent advances in biochemical screening assays and improved outcomes in HSCT-treated patients, a recent decision was made in 2024 to add KD to the Recommended Newborn Screening Panel (RUSP) [32–35]. With this new recommendation, there is expected to be an influx of newly diagnosed infants who are identified pre-symptomatically, and are thus candidates for HSCT or future disease-modifying therapies [25, 36]. However, given the paucity of published studies that investigate the impact of HSCT in relation to caregiver/patient reported outcomes, some families may remain hesitant to proceed with transplantation [32, 36, 37]. Such hesitation may delay treatment decisions in a rapidly progressive disease like IKD. In addition, the absence of caregiver/patient-reported outcomes is likely to be a detriment in discussions at the state level, as state legislatures debate whether to adopt the RUSP recommendation and invest in the infrastructure required for KD newborn screening (NBS) [38–40].

Furthermore, the goal of this study is to assess the impact of early HSCT on disease burden, quality of life, family function/dynamics, mental health, and financial stressors. We will elucidate key differences between children who were transplanted prior to the onset of symptoms to those who were either transplanted after the onset of symptoms or never transplanted. Our design involved collecting and analyzing data from qualitative parent interviews and quantitative data from surveys including the Leukodystrophy Quality of Life Assessment (LQLA) questionnaire [41]. This represents the largest investigation to systematically explore patient/family-centered experiences. This knowledge will be used to establish the patient’s voice in discussions involving policymaking related to NBS and help guide families when making the individualized decision of whether or not to proceed with transplantation.

## Methods

### Sample

The study sample was recruited in collaboration with KrabbeConnect, Partners for Krabbe Research, the United Leukodystrophy Foundation and individuals who had opted-in to be contacted for research participation through Engage Health’s EnCompass® database. Participants were recruited via email communication sent by the aforementioned groups and posts to social media sites. The content of emails and social media posts were pre-approved by the IRB. Eligible participants were individuals 18 years of age or older diagnosed with IKD/LIKD or were a caregiver of a person diagnosed with IKD/LIKD. IKD was defined as an infant with onset or expected onset of symptoms prior to 12 months of age. LIKD was defined as onset or expected onset of symptoms between 13 and 36 months of age [6, 7]. Parents of children who had passed away greater than 8 years from the time of providing consent were excluded from the interview portion in order to minimize recall bias. The goal was to recruit a total of 40 participants.

## Study design and procedures

This non-interventional patient reported outcomes (PRO) and observer reported outcomes (ObsRO) qualitative research study was approved by the WIRB (Western International Review Board- Copernicus Group) on January 30^th^, 2024. Upon study notification, interested participants were required to visit a HIPAA-compliant survey site, where they reviewed and signed an electronic consent, completed a questionnaire which included demographic information, information about the patient’s healthcare provider, the LQLA, and the EQ5D5L [42]. Of note, the EQ5D5L was not included in the final analysis due to a significant amount of missing data that limited its utility. The interview questions were designed based on current literature detailing KD natural history and HSCT outcomes, with input from clinicians experienced in the care of patients with KD [6, 7, 12, 23, 43, 44].

**Fig. 1:**
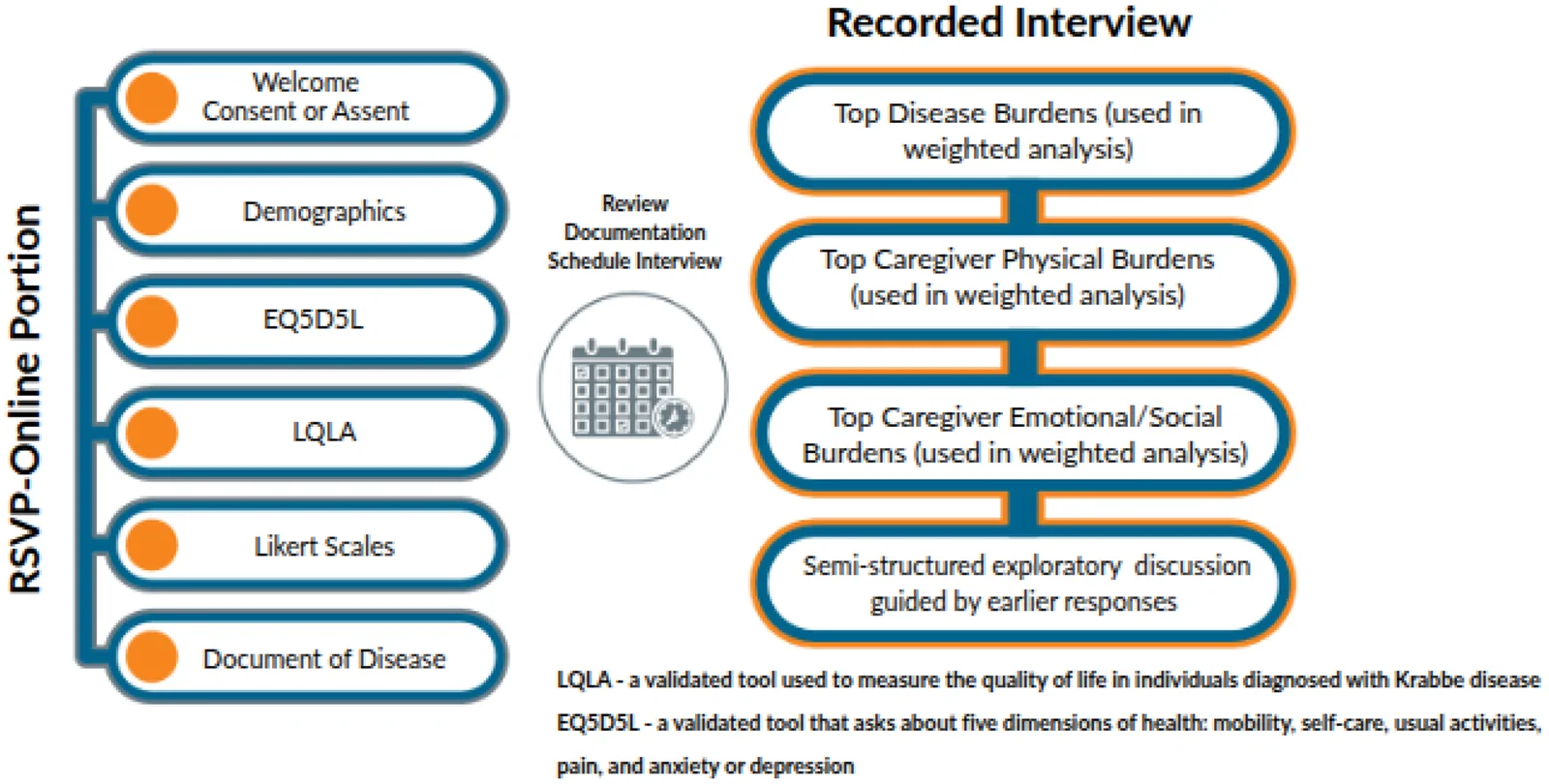
Summary of survey and interview structure

Potential participants completed an initial screening questionnaire inquiring about caregiver/disease burden, diagnostic data, and demographic information. Participants were required to provide ‘documentation of disease’ – specifically, a diagnostic or genetic testing report noting the pathogenic variants, GALC enzyme level, and/or psychosine levels. They were also required to provide an abbreviated clinical history. Participants were contacted directly in cases where further clinical or diagnostic clarification was necessary. Participants were excluded if unable to provide any of the three diagnostic/biochemical testing, or if the results ± clinical history were equivocal/not consistent with a diagnosis of IKD or LIKD. After pseudonymization, information was sent to the primary investigator (NAB), who approved the inclusion of each participant and assigned each to a category: IKD transplanted early, IKD non-transplanted/transplanted late, LIKD transplanted early, or LIKD non-transplanted/transplanted late. For the purpose of this paper, “transplanted early” is defined as an individual who was transplanted before symptom onset and “transplanted late” is defined as an individual transplanted after symptom onset. Each participant subsequently participated in a 1-to-1.5-hour telephone interview with staff who were trained in qualitative research. Questions were standardized with freedom for interviewers to delve deeper into topics if appropriate. Please refer to Fig. 1 for a visual representation of the survey workflow and interview structure. After completion of quality control, all study data was pseudonymized, removing any identifying information and assigning each patient a unique identifier.

## Statistical analysis

Thematic analysis of qualitative data obtained from open-ended interview questions was performed using MAXQDA 2020 (VERBI Software, 2019) in order to unpack caregiver experiences and disease burdens reported by patients and families impacted by IKD and LIKD. Quantitative data was collected using the LQLA and multiple Likert scales that assessed caregiver-reported burden. Results of the LQLA and Likert scales were analyzed using nonpaired t-tests. P-values of < 0.05 were considered statistically significant. Descriptive statistics were used to display demographic data and findings from the weighted analysis.

### Leukodystrophy quality of life assessment

The LQLA was administered to all participants as a quantitative tool for assessing patient disease burden. The LQLA, developed by Langan and colleagues, has been established as a valid and reliable survey for evaluating quality of life in IKD and LIKD. Notably, their 2019 study provided evidence of concurrent validity with the widely implemented Vineland Adaptive Behavior Scales [41]. The LQLA was therefore a well-suited tool for the current study. The LQLA consists of 43 questions divided into four domains (communication: 10 questions; daily living: 16 questions; social/family: 11 questions; Motor: 6 questions). Each participant survey was assigned an overall score and a subscore for each domain. A non-paired t-test was conducted in comparing overall scores and subscores. The following cohorts were juxtaposed against one another: A) IKD non-transplanted/transplanted late vs. IKD transplanted early; and B) LIKD non-transplanted/transplanted late vs. LIKD transplanted early.

### Weighted analysis

The weighted analysis was designed to collect open-ended responses that captured the most salient aspects of disease and caregiver burdens. Of note, the term “weighted” was selected as a descriptor for this portion of the interview because participants were able to “weigh” the detrimental effects of specific burdens in proportion to other burdens by assigning an individual score to each. The weighted analysis was conducted three times during the survey in order to evaluate the following three domains: 1) Disease burden on child; 2) Physical burden on caregiver; 3) Emotional/social burden on caregiver. Each of these domains were analyzed independently and summarized using descriptive statistics.

**Fig. 2:**
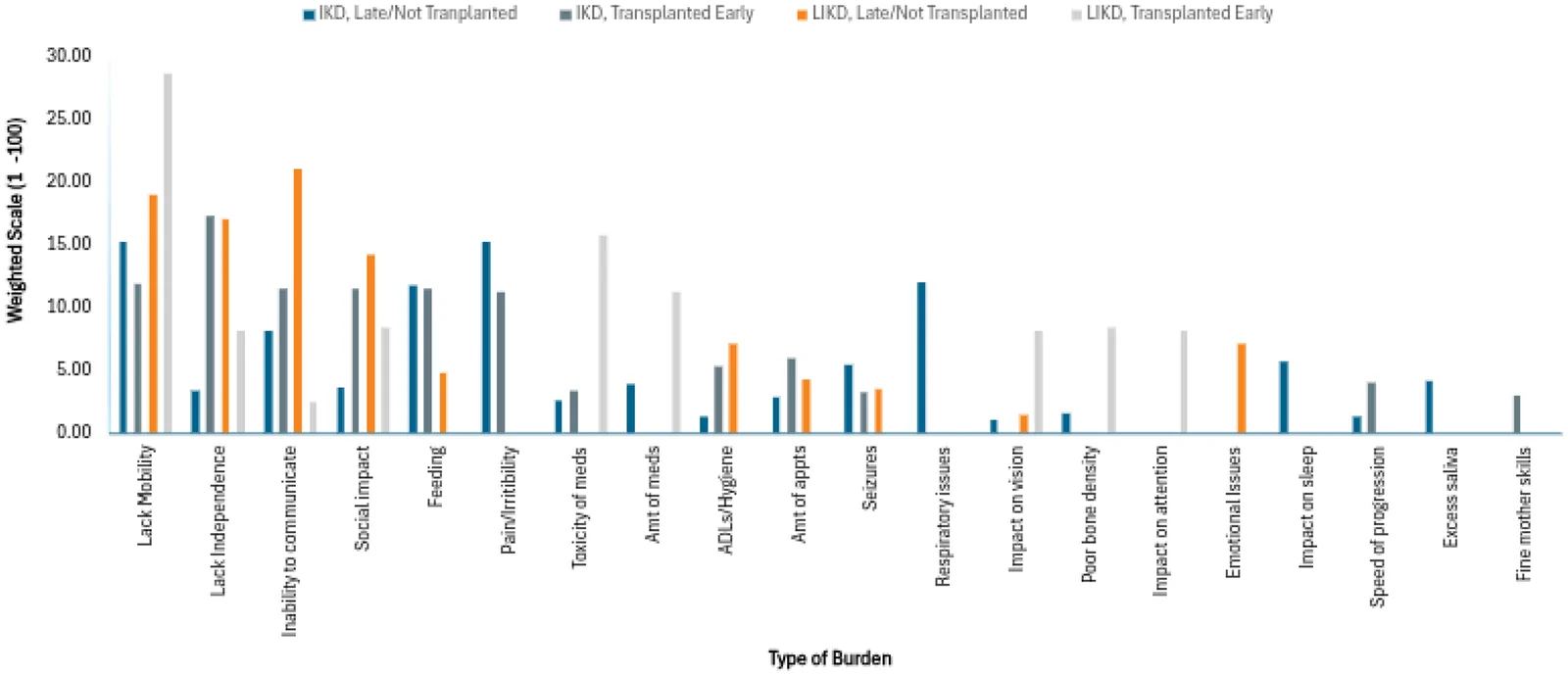
Weighted analysis - caregiver reporteddisease burden

Participants were allotted a total of 100 points for each of the domains. Within each domain, they were asked to describe the three most significant burdens. This could be done using a single word or multiple sentences. They were then tasked with distributing the 100 points across these burdens. Participants could use their discretion to distribute the 100 points as equally or unequally as they saw fit. Although the responses were open-ended, themes emerged, and each response was assigned to an overarching category. Total scores for each category were added and then divided by the number of participants in each cohort to obtain a mean score. Higher scores were indicative of more significant burdens. Results are depicted in Figs. 2, 4, and 6.

### Likert scale: caregiver burdens

**Fig. 3:**
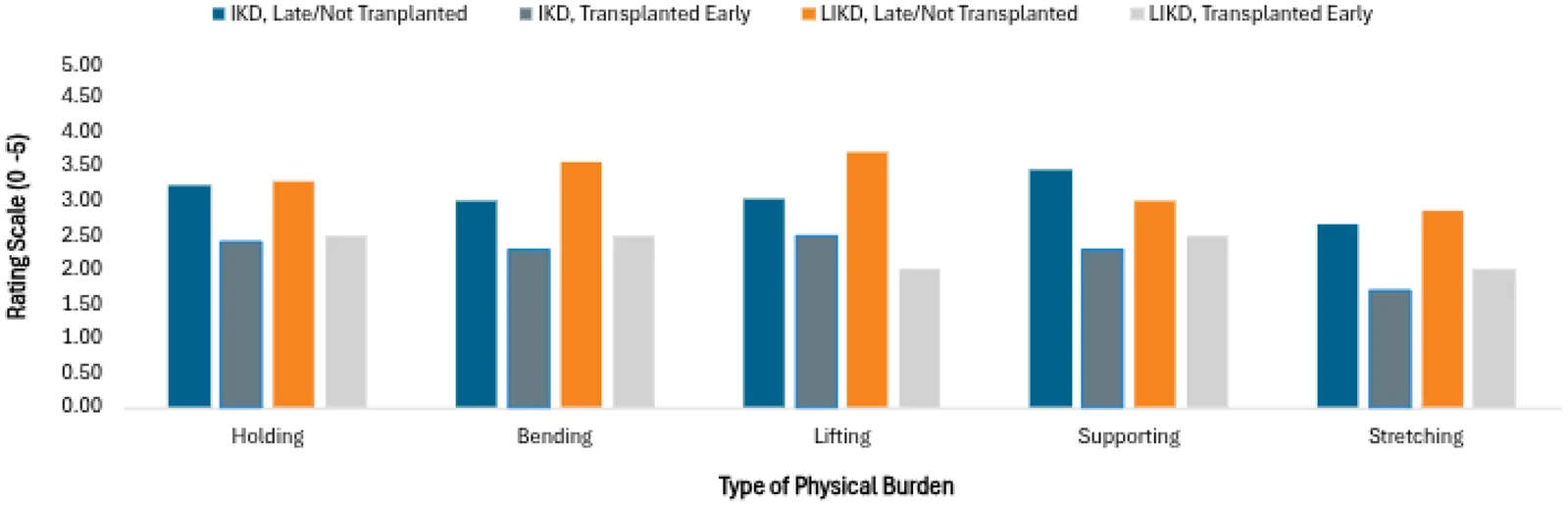
Likert scale - physical burdens experiencedby caregiver

To investigate caregiver well-being, a Likert-scale was utilized posing various questions that assessed the degree of physical and emotional/social burdens. The scale ranged from one to five (1 = Not at all; 2 = A little; 3 = Somewhat; 4 = Very; 5 = Extremely). A non-paired t-test was conducted for individual questions to detect statistically significant differences between the transplanted early and non-transplanted/transplanted late groups. Results are displayed in Figs 3 and 5.

## Results

### Demographics

The study consisted of interviews with 40 participants who completed the survey and interview. There were an additional three participants who only completed the survey but were later excluded from the full interview. These three patients were excluded because they either died more than 8 years ago (*N* = 2) or completed the survey after the interview portion of the study was closed (*N* = 1). The 40 patients meeting inclusion criteria and completing both the survey and interview were categorized as follows: IKD non-transplant/transplanted late (*n* = 19); IKD transplanted early (*n* = 10); LIKD non-transplant/transplanted late (*n* = 7); LIKD transplanted early (*n* = 4). Most participants resided in the US (*n* = 36), followed by Honduras (*n* = 2), Spain (*n* = 1), and Australia (*n* = 1). In nearly all cases (97.7%), the participant was a parent of the individual diagnosed with KD, and of those, 90.7% were the mother. One interview consisted of a father and patient interviewing together. There was one set of siblings from the same family included, with the same parent participating in both interviews. There were 23 (57.5%) male patients and 17 (42.5%) female patients.

61.8% of children were living at the time of the interview. Those who were deceased were most often from the IKD non-transplant/transplanted late group, with 63.6% of individuals in that group deceased at the time of interview (mean age of death: 35.6 months; range 0 to 97 months). While there were 2 deaths in the IKD transplanted early group, both were due to transplantation complications, and both had undergone a full-conditioning regimen. The mean age of the patients at the time of interview was 67.3 months, 74.6 months, 120 months and 219 months for IKD non-transplant/transplanted late, IKD transplanted early, LIKD non-transplant/transplanted late and LIKD transplanted early, respectively (Table 1).

**Table 1:** Demographics (*N* = 40)

Cohort	N=	Deceased?	Mean age	Mean age of diagnosis	Diagnosed with NBS?
IKD Late/Non-transplant	19	14 (64%)	67.3 mo	7.1 mo	1 (5%)
IKD Early transplant	10	2 (20%)	74.6 mo	0.3 mo	9 (90%)
LIKD Late/Non-transplant	7	0 (0%)	120 mo	29.7 mo	1 (14%)
LIKD Early transplant	4	0 (0%)	219 mo	13.8 mo	0 (0%)

Table 1 depicts the number of patients included in each cohort. “Mean age” represents the age (in months) of patients at the time their caregiver was interviewed or the age when they died, when applicable. The table also displays the number of deceased patients, number of patients diagnosed via newborn screening, and the mean age at the time of diagnosis (in months) for the four distinct cohorts

### Diagnostic testing

Diagnostic biochemical or genetic testing was collected for all participants. Of the 40, 19 (47.5%) had GALC enzyme levels, 21 (52.5%%) had genetic testing results, and 10 (25%) had psychosine levels. Of the ten patients with psychosine levels, nine (mean: 65.6 nmol/L; range: 14–149 nmol/L) were diagnosed with IKD and one (2.2 nmol/L) with LIKD.

### Timing of diagnosis, transplantation, and symptom onset

The average age of diagnosis for IKD non-transplanted/transplanted late was 7.1 months compared to 0.3 months for the IKD transplanted early group. The LIKD non-transplanted/transplanted late group averaged 29.7 months of age at the time of diagnosis compared to 13.8 months for the LIKD transplanted early group. The average delay between disease onset and diagnosis was 4.7 and 4.3 months for non-transplanted/transplanted IKD and LIKD patients, respectively. In the case of pre-symptomatic diagnosis, it was due either to positive results on NBS (*N* = 11), or the presence of known family history (*N* = 7). Concordantly, the most common reason for early transplantation was NBS (71.4%) followed by family history (28.6%). NBS was particularly important for the IKD transplanted early group, in which 9/10 (90%) occurred due to routine NBS at birth.

Of the IKD non-transplanted/transplanted late, 7 (31.8%) were transplanted late and 15 (68.2%) were never transplanted. In the LIKD non-transplanted/transplanted late cohort, 5 (71.4%) were transplanted late and 2 (28.6%) were never transplanted.

#### Disease burden

For the IKD transplanted early cohort, analysis of the LQLA revealed several statistically significant differences in overall score and subscores for communication and daily living compared to their non-transplanted/transplanted late counterparts (Table 2a). Similarly, caregivers of LIKD patients who were transplanted early reported statistically significant better overall scores, as well as higher scores in motor skills and daily living (Table 2b)

**Table 2a:** IKD LQLA

Domain	Early transplant	Late/Non-transplant	P-value
Overall	Mean 30.7; Range 23–38	Mean 20.6; Range 13–27	p < 0.001**
Communication	Mean 8.7; Range 5–10	Mean 4.8; Range 1–10	p < 0.001**
Daily Living	Mean 11.7; Range 9–14	Mean 8.8; Range 5–12	p < 0.001**
Social/Family	Mean 6.7; Range 4–9	Mean 5.8; Range 3–9	p = 0.089
Motor	Mean 2; Range 0–5	Mean 1.2; Range 0–4	p = 0.089

Table 2a depicts the results of the LQLA for patients with infantile onset. The asterisks (**) indicate categories where there was a statistically significant difference between the early transplant and Late/Non transplanted cohorts

**Table 2b:** LIKD LQLA

Domain	Early transplant	Late/Non-transplant	P-value
Overall	Mean 33 Range 26–40	Mean 25.3; Range 15–29	p = 0.022**
Communication	Mean 8; Range 6–10	Mean 8.3; Range 5–10	p = 0.431
Daily Living	Mean 12.8; Range 11–14	Mean 9.4; Range 4–13	p = 0.032**
Social/Family	Mean 7; Range 6–10	Mean 5.7; Range 3–8	p = 0.103
Motor	Mean 4.8; Range 3–6	Mean 1.9; Range 0–5	p = 0.007**

Table 2b depicts the results of the LQLA for patients with late-infantile onset. The asterisks (**) indicate categories where there was a statistically significant difference between the early transplant and Late/Non transplanted cohorts

Turning to the weighted 100 point analysis, the two most significant disease burdens reported by caregivers of IKD children in the non-transplanted/transplanted late cohort were lack of mobility/movement and pain. These were closely followed by respiratory issues. In comparison, those who were transplanted early reported limitations in independence/normal life as the largest burden, with other notably impacted areas including feeding, communication, socialization, mobility and pain. As for the LIKD cohorts, the most significant disease burden was lack of mobility/movement for patients transplanted early and inability to communicate for those non-transplanted/transplanted late (Fig. 2).

## Caregiver burden

### Physical burden

Participants were asked to rate the degree of physical stress related to various tasks and activities using a Likert scale. The scale ranged from one to five, with one being the least burdensome and five being the most. Analysis revealed that caregivers of IKD children who were transplanted early benefitted from statistically significant less physical stress from holding (*p* = 0.046), supporting (*p* = 0.018), and stretching (*p* = 0.033). While there was a tendency for higher burden scores in the LIKD non-transplant/transplanted late group, statistical significance was not met for any of the questions (Fig. 3).

**Fig. 4:**
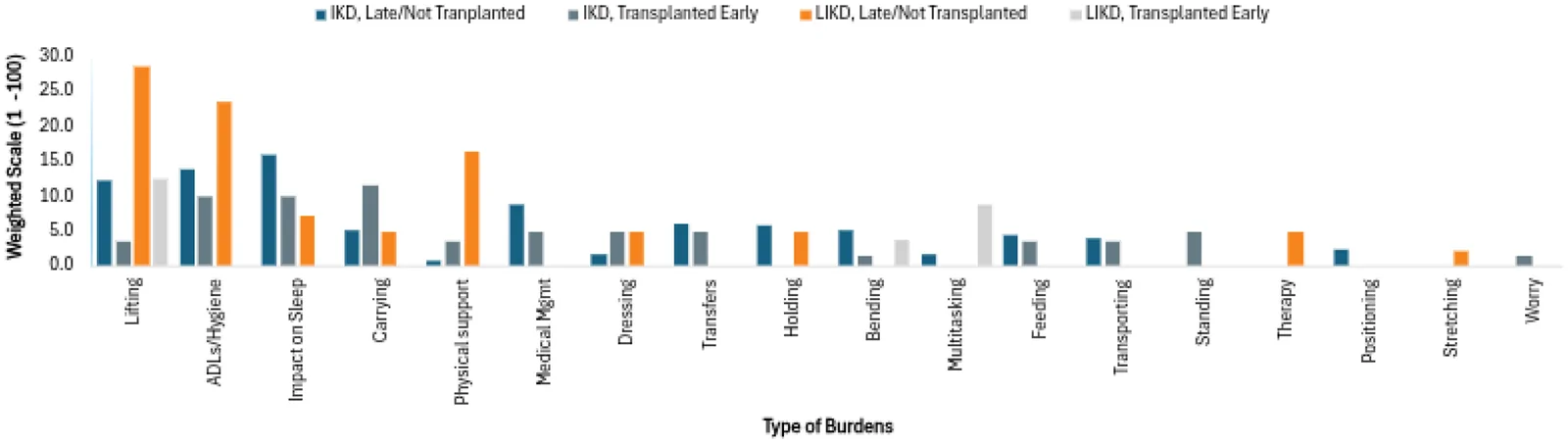
Weighted analysis - physical burdensexperienced by caregiver

Study participants were also asked to describe the most burdensome physical aspects of caregiving in an open-ended manner using the 100 point weighted analysis. For IKD non-transplanted/transplanted late, caregivers designated impact on sleep as the largest burden. Other heavily impacted areas for IKD non-transplanted/transplanted late included impaired activities of daily living (ADLs) and lifting. As a whole, weighted scores were more evenly distributed across multiple categories in the IKD transplanted early group with carrying being the largest burden. However, there was considerable variability in the IKD transplanted early group, with caregivers of older children often assigning higher scores to physically demanding tasks than those with younger children. LIKD patients who were non-transplanted/transplanted late indicated lifting, ADLs, and physical support as the largest burdens. Limited data was available for the LIKD transplanted early group (Fig. 4).

### Emotional/Social burden

**Fig. 5:**
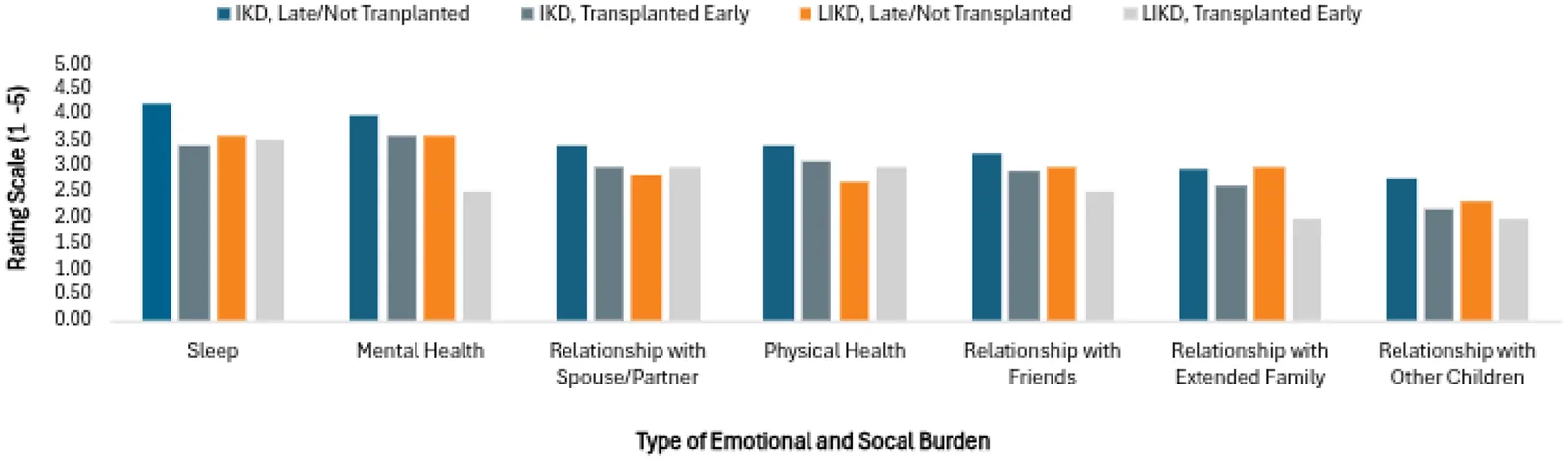
Likert scale - emotional and social burdensexperienced by caregiver

Participants were asked to rate the degree of impact on various social and emotional domains of daily life using a Likert scale. As above, the scale ranged from one to five, with one being the least impacted and five being the most. For the IKD phenotype, analysis revealed a statistically significant improvement in the transplanted early group for sleep (*p* = 0.043), mental health (*p* = 0.049), relationship with spouse/partner (*p* = 0.049), and relationship with other children (*p* = 0.048). While there was a tendency for higher scores when rating relationships with friends and extended family and physical health, these did not meet statistical significance. More variability was seen in the LIKD groups, possibly due to the smaller sample size, however, no categories reached statistical significance (Fig. 5).

**Fig. 6:**
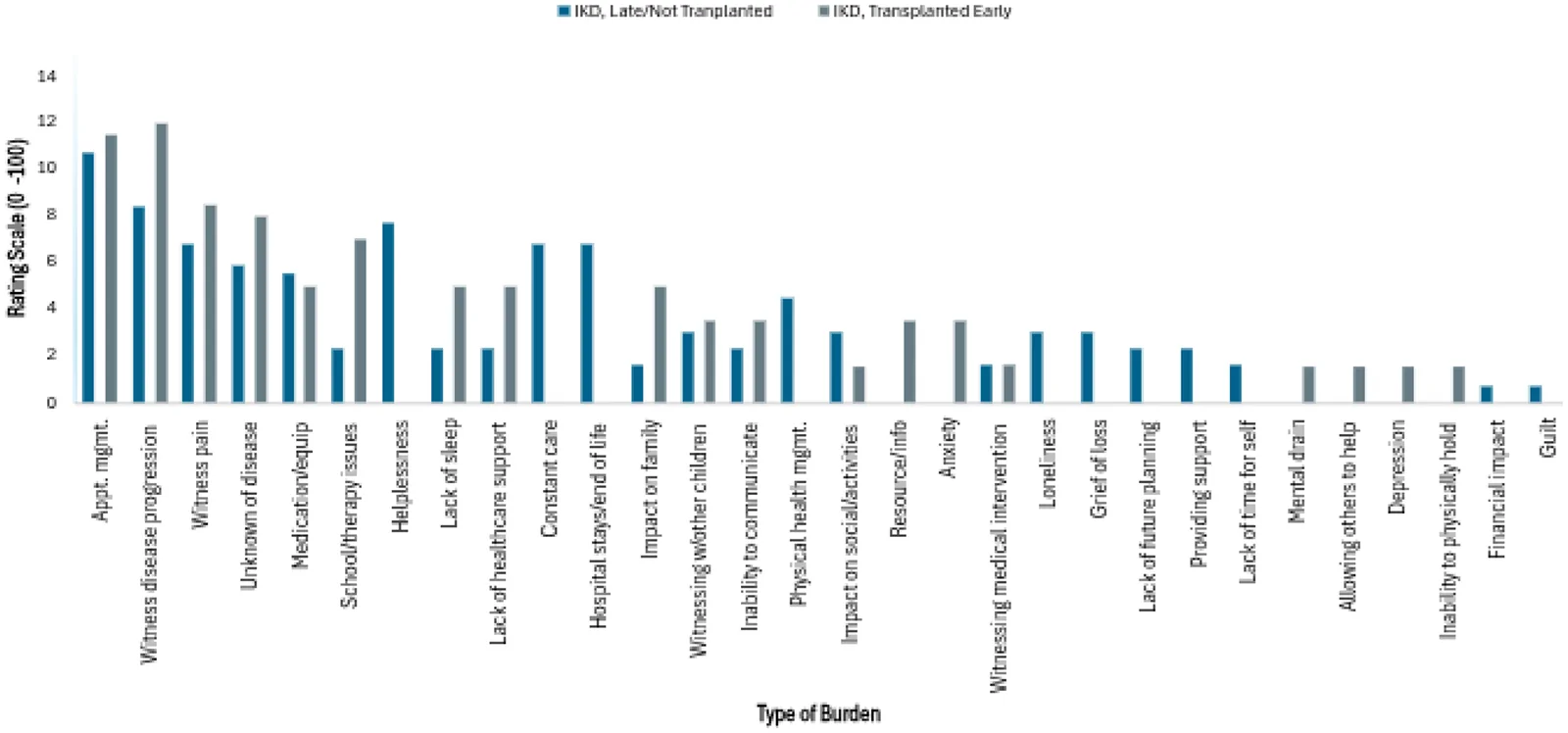
Weighted analysis - emotional and socialburdens experienced by caregiver (IKD only)

The results of the open-ended emotional/social 100 point weighted analysis for IKD patients are depicted in Fig. 6. Appointment management and witnessing disease progression were the top two burdens for both IKD transplanted early and non-transplanted/transplanted late. Other burdens with considerable overlap included witnessing pain, medication and equipment access, and the unknowns of disease. In terms of differences, IKD non-transplanted/transplanted late caregivers struggled more from hopelessness, feeling the need to be constantly vigilant, extended hospital stays and end of life care, loneliness, and grief. In comparison, IKD transplanted early caregivers reported a greater impact on family, issues with school or therapy, lack of support from the healthcare system, limited information on outside resources, and anxiety.

### Time, educational, and financial burdens

Caregivers in the IKD non-transplanted/transplanted late were the most likely to report missed educational and occupational opportunities (59.1%), followed by LIKD non-transplanted/transplanted late (42.9%). In comparison, only 30 and 25% of IKD and LIKD transplanted early reported missed opportunities, respectively. Regardless of subtype, all families averaged at least 40 hours per week providing care for their child. The mean number of hours per week spent providing care were as follows – IKD transplanted early: 107 hours, IKD non-transplanted/transplanted late: 117 hours, LIKD transplanted early: 42 hours, LIKD non-transplanted/transplanted late: 76 hours

Annual unreimbursed medical expenses were greatest for those in the IKD (mean: $10,945) and LIKD (mean: $5,645) non-transplanted/transplanted late groups. Severe caregiver physical injuries were relatively infrequent among all groups but highest in the IKD non-transplanted/transplanted late group (13.6%).

### Irritability

Of the whole cohort, 63% of participants experienced intense irritability episodes. Prevalence was very high (94.7%) in the IKD non-transplanted/transplanted late group but much less common (20%) for IKD transplanted early (*p* < 0.01). Median age of irritability onset was 3 months (range: 0 to 10 months) for IKD patients and was often the first sign of disease. Irritability was less common for LIKD patients (non-transplanted/transplanted late: 71.4%; transplanted early: 0%) with median age of irritability onset of 30 months (range: 14 to 34 months).

There was a multiple month delay between irritability identification and diagnosis in all patients not identified through NBS. Almost half (44%) of caregivers reported constant frequency/duration while awake and an associated high-pitched cry. An overwhelming majority (85%) reported increased tone and rigid extremities during episodes. Caregivers reported gabapentin and baclofen as the most effective medications. Medications targeting gastrointestinal pathophysiology, such as dicyclomine and simethicone, were ubiquitously ineffective, with no caregivers reporting benefit or improvement with this class of medications.

### Desired outcomes for new therapies

At the end of the interview, participants were asked to imagine that there was a new drug for KD intended to slow or halt disease progression and to describe their three ideal or desired outcomes. When comparing the desired outcomes, caregivers in both IKD groups were most concerned with having an impact on gross motor skills or mobility. Caregivers of LIKD patients who were transplanted late/not were most concerned with communication, while LIKD transplanted early provided responses with nonspecific improvements such as ‘modifying disease’ and ‘general symptom management.’

### Illustrative caregiver remarks

In addition to the extensive quantitative data provided above, we collected a large array of specific quotations and have selected multiple caregiver remarks that illustrate different struggles involved in caring for a child with KD. Responses involving four categories were chosen and are depicted in Appendix 1. The four categories were as follows: 1) Missed educational opportunities due to caregiving for a child diagnosed with KD; 2) Impact of caring for child with KD on spousal relationship; 3) Impact on caregiver sleep due to having child with KD; and 4) Ability for the affected child to interact/socialize with others. Note that the selected remarks are only a small portion of the larger dataset.

## Discussion

To our knowledge, this study represents the largest investigation to systematically explore patient/family-centered experiences of HSCT in KD and provides convincing evidence that HSCT improves quality of life and reduces caregiver burden in IKD. This is commensurate with an extensive body of previous publications that have established strong clinical evidence demonstrating the efficacy of pre-symptomatic HSCT as a disease-modifying therapy in both IKD and LIKD [23–29]. Moreover, despite its well-established treatment effect, there has been a dearth of data on the outcomes of HSCT on quality of life from the perspective of the caregiver up to this point. This glaring gap in the literature was underscored by a 2024 systematic literature review conducted by Koto and colleagues, which found no publications explicitly reporting on first-hand experiences caring for a loved-one with KD [31]. Since then, there has only been one study attempting to address the gap. Although the authors’ efforts produced valuable information, that cohort consisted of only 3 patients (1 LIKD, 1 juvenile, and 1 adult) and did not include data on HSCT [30].

Furthermore, our study is critically important given its design that permits for a comprehensive assessment of the impact of HSCT on quality of life and caregiver burden, in which burden is defined as the multidimensional response to physical, psychological, emotional, social, and financial stresses associated with the caregiver experience. The overall structure of the interview and subsequent analysis provides a basis for delineating differences within three main categories of burdens: 1) Disease burden on the patient; 2) Physical burden on the caregiver; and 3) Emotional/social burden on the caregiver. While data is limited in the LIKD group due to the small sample size, our IKD groups are much larger and provide a robust foundation from which to draw conclusions. Therefore, the majority of the following discussion focuses primarily on comparing disease and caregiver burdens between IKD transplanted early against IKD non-transplanted/transplanted late and will only briefly touch on the LIKD cohorts.

For the purpose of this discussion, we will consider the data acquired on disease burden as a proxy for patient quality of life, as perceived by family members. As expected, results of the LQLA demonstrated significantly less disease burden in both IKD and LIKD groups who were transplanted early (Table 2a, 2b). While results of the weighted analysis revealed significantly impaired quality of life for both IKD transplanted early and IKD non-transplanted/transplanted late, there was a general shift in the features of disease burden, with non-transplanted/transplanted late patients unable to overcome basic detriments to quality of life such as respiratory distress, immobility, and pain. In comparison, the IKD transplanted early group were better equipped to transcend these minimal requirements for quality of life but continued to suffer markedly from impacts on communication, socialization, ADLs, and complex medication regimens (Fig. 2).

We next turn to evaluating the extent of physical burdens imposed directly upon the caregiver. Analysis of responses to the Likert scale and weighted analysis both demonstrated a tendency towards decreased bodily stresses experienced by caregivers of IKD patients transplanted early compared to their non-transplanted/transplanted late counterparts, though this varied depending on the task. For instance, statistical significance was met for holding, supporting, and stretching but not for bending or lifting on the Likert scale (Fig. 3). Nevertheless, there are some meaningful differences between the Likert scale and weighted analysis that allow for a more nuanced interpretation. Specifically, the Likert scale, with its closed-ended categories and ordinal rating scale, is well-suited for providing general information on each group without being significantly skewed by outliers. In comparison, the weighted analysis allowed for more individualized open-ended responses and enabled caregivers to disproportionately assign scores to aspects of their daily lives causing them the most physical stress. By doing so, we found that all IKD non-transplanted/transplanted late caregivers experienced a large quantity of physical burden and typically struggled with similar physical stressors. This contrasts with caregivers belonging to the IKD transplanted early cohort, where the weighted analysis suggests a greater degree of variability in the amount of physical burden as well as disparities in the primary source of the burden (Fig. 4). This is best exemplified by responses falling under the ´carrying´ category, in which some caregivers assigned a high score and others did not mention it (other examples include transfers, dressing, and standing). Diving into this intra-group difference further, we found caregivers of transplanted early children often enjoyed relatively little physical burden in the initial years when compared to the non-transplanted/transplanted late group but began to experience progressively more physical stress as their children continued to grow. Along these lines, participants who assigned a higher weighted score to ´carrying´ were generally caring for older and larger children than individuals with a lower score (Fig. 4). This is consistent with current knowledge of the disease-modifying effects of HSCT, in which gross motor skills benefit minimally from HSCT despite improvements in other neurodevelopmental domains, thereby causing increased physical demands on those assisting with daily care as patients age. It also aligns with responses to what IKD caregivers would want out of future therapies, thus highlighting the limitations of HSCT and the need to continue investigating novel interventions such as gene therapy or small molecules [45–51].

Finally, we cannot overlook the emotional and social impact on those caring for children with KD. Reviewing the raw responses from the unstructured portion of the interview, discussions frequently focused on how caring for a child with KD impacted their mental health, marriage, relationships with other children/extended family/friends, missed occupational and educational opportunities, and financial status (Appendix 1). Such social and emotional burdens were prevalent irrespective of phenotype or treatment status, but there were again meaningful differences between the groups. For example, when asked directly about certain high burden areas on the Likert scale, caregivers of IKD transplanted early responded with statistically significant less burden in sleep, mental health, relationship with spouse/partner and relationships with other children (Fig. 5). When the open-ended responses were analyzed using the 100 point weighted analysis, we found that both IKD caregivers suffered immensely and relatively equally from witnessing their child´s disease progression, witnessing their pain, and difficulties with management of medical appointments. However, a more nuanced interpretation of results from the weighted analysis suggests that the types of burdens begin to diverge in other categories as caregivers contemplate the effects on their daily life. Particularly, caregivers in the IKD non-transplanted/transplanted late group suffered more from feelings of constant vigilance, extended hospital stays, and end-of-life care/discussions, which subsequently manifested into higher reports of mental distress in the form of loneliness, helplessness, guilt, and lack of time for oneself (although it should be noted that anxiety was the one exception that scored higher in the transplanted early group). In comparison, our IKD transplanted early group did not frequently cite these severe and direct impacts on emotional health and instead focused more on the social impacts, often citing burdens on family dynamics, issues with school or therapy, lack of support from the health care system, and limited information on outside resources (Fig. 6). From a separate portion of the interview, we also found higher amounts of unreimbursed financial costs and missed educational/occupational opportunities in the IKD non-transplanted/transplanted late cohort. Moreover, the significance of these findings cannot be overstated, as studies in other neurologic and genetic diseases have provided a well-established correlation between psychological health and perceived caregiver burden, with higher amounts of stress, anxiety, depression, and poor social connectedness associated with a higher degree of perceived burden from caregiving responsibilities and corresponding reduced quality of life for both patients and caregivers [52–56].

### Implications

As of October 2025, there are currently fifteen states actively screening for IKD. This is an increase from eleven states at the time KD was first added to the RUSP in July 2024. If adoption of NBS continues at this rate, it will still be years before most US states pass NBS legislation and develop the corresponding infrastructure required for large-scale screening programs. By providing a voice for patients and caregivers, the data presented here provides a wealth of information and is an invaluable resource for policymakers at the state level who are debating whether to add KD to their NBS panel with the intention of facilitating early diagnosis and treatment. As such, the efficacy of NBS is exemplified by the fact that 90% of the children in the IKD transplanted early group were identified by NBS.

Assuming the number of screening states does continue to expand, a growing population of families will be presented with the opportunity of pursuing early treatment. Given the urgency to make a decision on transplantation prior to disease progression, families may often struggle with the issue of feeling like they have insufficient information to make an educated determination of the risks and benefits of such an intensive treatment. While knowledge gaps will always be inevitable, having access to the collective wisdom of the caregivers interviewed here may enable future caregivers to approach the life-altering decision of whether to pursue transplantation with a greater degree of confidence and provide enlightenment regarding what to expect. Thus, our findings should aid in the amelioration of unnecessary treatment delays and minimize the potential for parental guilt and remorse after missing the critical window for early transplantation. Alternatively, it may help other caregivers better determine if transplantation lay outside their goals of care.

### Limitations

A limitation of this study was the relatively small sample size, particularly in the LIKD group, as well as an uneven distribution between transplanted early and non-transplanted/transplanted late cohorts. However, the skew between IKD and LIKD is expected and closely mirrors the estimated distribution of each phenotype within the general population, where 85–90% of cases are IKD and 10–15% are LIKD. Another key limitation was the cross-sectional methodology of the interviews with absence of longitudinal follow up. The nature of the interviews also introduced the possibility for recall bias when discussing aspects of the child’s disease that occurred months or years prior to the interview itself. In addition, there was a mild geographic preference for the Midwest based on KrabbeConnect’s headquarters located in Minnesota. Along these lines, there was an IKD transplanted early subgroup with a trend towards younger age due to a wave of new diagnoses and recruitment after Minnesota adopted NBS in February 2024. Lastly, caregivers often did not have immediate access to diagnostic testing (psychosine, genetics, enzyme levels), leading to delays in recruitment as we awaited confirmation of diagnosis.

## Conclusions

This is the largest investigation to systematically explore patient/family-centered experiences and provides convincing evidence that HSCT improves quality of life and reduces caregiver burden in IKD. In general, early transplanted families benefitted from improved familial and social relationships, less missed educational opportunities, less financial strain, better sleep, and less time providing care. Factors protecting against caregiver burden included positive coping strategies, close spousal bonds, and finding positive aspects of caring. Maintaining social networks was also important for promoting positive aspects of caregiving and reducing perceived burden. Although there was variability between individual responses, parents in the IKD transplanted early group often suffered less physical stress, though this trend started to reverse as transplanted children grew older. This knowledge will prove invaluable to states debating whether to add KD to their NBS panels and for caregivers engaging in transplantation decision-making.

## Electronic supplementary material

Below is the link to the electronic supplementary material.

Supplementary Material 1: Appendix 1: Illustrative caregiver remarks

## Data Availability

The raw data for the manuscript is currently being used in other studies. However, the data is available upon request.

## Abbreviations

ADLs: Activities of daily living
GALC: Galactosylceramidase, an enzyme crucial for breaking down certain fats, specifically galactolipids, which are important to the nervous system and kidneys
HSCT: Hematopoietic Stem Cell Transplantation, a medical procedure that involves infusing hematopoietic stems cells to replace or reconstitute the hematopoietic system
IKD: Infantile Krabbe disease, onset of symptoms 13–36 months
KD: Krabbe disease
LIKD: Late infantile Krabbe disease, onset of symptoms before 12 months
LQLA: Leukodystrophy Quality of Life Assessment, a validated quality of life tool that includes questions relevant to the extreme burden of disability and medical problems experienced by those diagnosed with a leukodystrophy such as Krabbe disease
NBS: Newborn screening, a public health program that screens newborns for certain genetic, endocrine, and metabolic disorders as well as hearing loss and critical congenital heart defects
ObsRo: Observer-reported outcomes
PRO: Patient-reported outcomes
RUSP: Recommended Uniform Screening Panel, a list of disorders that the Secretary of the Department of Health and Human Services recommends for states to screen as part of their state universal newborn screening programs
WIRB: Western Institutional Review Board
