# Correction: Caregiver-reported disease burden in Krabbe disease: evaluating outcomes of hematopoietic stem cell transplantation

**DOI:** 10.1186/s13023-026-04480-6

**Published:** 2026-08-24

**Authors:** Nicholas Alexander Bascou, Skyler Jackson, Patti Engel, Anne Melchior, Paul Orchard, Stacy Pike-Langenfeld

**Affiliations:** 1 Department of Neurology, Johns Hopkins University, 1800 Orleans Street, Baltimore, MD USA; 2 Krabbe Connect, Rosemount, MN USA; 3 Engage Health, Eagan, MN USA; 4 Division of Pediatric Bone Marrow Transplantation, University of Minnesota, Minneapolis, MN USA

**Correction: Orphanet Journal of Rare Diseases (2026) 21:36**

10.1186/s13023-025-04176-3

Following publication of the original article [1], we have been notified that Appendix 1 was mentioned in the article text, but not tagged.

The original article was updated.

## References

[1] Bascou NA, et al. Caregiver-reported disease burden in Krabbe disease: evaluating outcomes of hematopoietic stem cell transplantation. Orphanet J Rare Dis. 2026;21:36. 10.1186/s13023-025-04176-3. 10.1186/s13023-025-04176-3 PMC12870309 41501923

