# Supplementary material for: Caregiver-reported disease burden in Krabbe disease: evaluating outcomes of hematopoietic stem cell transplantation
Source: Orphanet J Rare Dis. 2026 Jan 7;21:36. doi: 10.1186/s13023-025-04176-3 (PMC12870309; doi:10.1186/s13023-025-04176-3)
Supplement: Supplementary file 1 [file 13023_2025_4176_MOESM1_ESM.pdf]

APPENDIX 1: Illustrative caregiver remarks

Responses to: Have you missed an educational activity or opportunity due caring for a person diagnosed with Krabbe disease?	Phenotype, Transplant status	Implications
Yes I wanted to go back to school to better my education and only made it one semester due to <name redacted> health declined and needed surgery. Not able to work so the financial stress all on my husband not being able to be there for my other children's sports plays school events.	IKD, Transplanted early	Dropped out of school
Yes. I can't travel with <name redacted> or just feel overwhelmed and scared due to his weight and hardships to get him around and also worry he will get sick. I miss all of the Symposiums and Galas for Krabbe Families that I would love to attend. I also cannot find anyone willing or able to spend the night with him.	IKD, Not transplanted/transplanted late	Unable to attend Krabbe-related opportunities
Yes many milestones in my other children's lives have been affected by myself being a full time caregiver	IKD, Not transplanted/transplanted late	Unable to participate in other children's milestones
Yes. I have spent the last 7 years caring for my son and other children at home. I have had employment opportunities I have had to turn down as I need to be home for his care.	IKD, Not transplanted/transplanted late	Missed employment opportunities
I dropped out of college to care for <name redacted> full-time. She required 24/7 care. If I didn't stay home to care for her she would need to be at a facility that could provide around-the-clock care. After her diagnosis, she was on palliative and supportive care until she died.	IKD, Not transplanted/transplanted late	Dropped out of college
I had to miss several work related job trainings due to being at the hospital for several months with <name redacted>.	IKD, Not transplanted/transplanted late	Missed training opportunities
Yes, left work to care for child during transplant and recovery	IKD, Transplanted early	Left job
**Responses to: Have you, as a caregiver, encountered challenges with your spousal relationship due to caring for someone with Krabbe disease?**	**Phenotype, Transplant status**	**Implications**
When you go through something horrific together, it has almost made us stronger, when you're always looking for the silver lining weirdly that is one of them, forces you to be a better team because you are balancing a lot of things you shouldn't have to	IKD, Transplanted early	Stronger relationship
We don't set time aside for each other, can't remember the last date night because I am always tired when he gets home, I am up all night and we don't have time for each other	IKD, Transplanted early	Lack of intimate time
Both good and bad- has brought us closer especially during transplant process, we are less laid back than we were, a little less patience with each other, conversations about expanding our family or not or second guessing is not something we talk about- has strengthened in some areas but has brought up other things that wouldn't have had to discuss	IKD, Transplanted early	Strengthened some areas and weakened others
I have always been the caregiver for our son, even when I worked as a phlebotomist, wasn't that different that I was the only one there, wasn't receiving calls- only once in a while. It's hard when you realize your partner wasn't there, he never even called my son, the diagnosis opened my eyes to realize really what was going on, it's a big weight right on the relationship especially when you see your significant other isn't present for whatever reason, being scared maybe. The ICU doctors didn't tell us she might pass away, husband was almost home and chose not to come back. The day it was confirmed that she didn't have much longer, I made all those decisions, dad wasn't even there, I called him and told him she wasn't going to make it and he needed to get there, after I talked to him the providers called him and still he didn't come. Some bind together during these time but some don't, for us it went the other way, things are not the best now- I came back with an open mind, I love my children and him, was willing to work on things, like we are just roommates now basically, sometimes you have to think if you have the emotional capacity to deal with that, focusing on healing my son and I- that hymen phase of my daughter being gone has hit, that mentality of 'we're good everything is fine' - my son and I have had a difficult week so we are just working on our emotions, don't have time to deal with grown up things	IKD, Transplanted early	Navigated a lot without spouse at the hospital, continue to work on relationship

The diagnosis and being away for transplant tied into my divorce, we both handled it a lot differently, he was away from him for 9 months while he got the transplant, wasn't there during a lot of it and didn't understand a lot of it. He was shell shocked when he got back, couldn't handle all the changes in medical care. I was traumatized and so tired- had lived in a hospital for 9 months and needed help, wasn't getting help and he shut himself off with video games. We coped a lot differently and still do to this day. For me I see my son as a blessing and for him it's more like he's going to die and doesn't want to think about the good stuff- but you can't think about just the good stuff. I wouldn't say the diagnosis was the only reason but pushed us towards that. We had issues before that - been together 6 years and only been married 1 year.	IKD, Not transplanted/transplanted late	Tied into the divorce
Yes- due to the space that equipment requires we sleep in different rooms and the spouse has little/no tolerance to not sleeping, gets moody and irritable without sleep which is difficult with the constant interruptions	IKD, Not transplanted/transplanted late	Sleep in separate rooms
It has impacted all areas of our life; we don't know when things will happen. We have 2 other kids and I cant spend as much time with them. Same with husband, did a lot of counseling. Friends and family are good support system but they try to help come along to therapy and they take turns driving, they really try to help where they can. Friends have helped to raise funds to help with medical costs.	LIKD, Not transplanted/transplanted late	Lack of time with other kids
Overall we have grown a lot stronger, we have moments we both deal with this disease very differently, I push it out of my mind and he analyzes over and over - we balance each other out but have moments where, like during transplant, wasn't sure our marriage would make it. You are in a hospital for 6 months, the amount of anger this disease brings gets taken out on your spouse. Thankfully we have gotten stronger and it has made us where there's no doubt, I could do anything without him, he understands everything related to this disease.	LIKD, Not transplanted/transplanted late	Manage emotions differently but continue to find strength to remain together
It has definitely brought us closer in some ways, we can really go through hell together, living in the hospital for treatments was awful, my husband has always been very supportive and when we got dx they were going through all the options and we had to go to Pittsburgh and my husband said we went through a lot to have him- went through IVF to have him, wanted to do everything we could to keep him, when we were at Pittsburgh, while he was undergoing anesthesia for the first time I was thinking I just was going to be in a hospital room by myself and he just said you have to and you can, he believes I am way more capable than I am, it did hurt our intimacy, last time we went out to dinner was last time the baby was in the PICU, since being home we haven't had a date so we need to start prioritizing that again	LIKD, Transplanted early	Lack of intimacy
**Responses to: How does the individual with Krabbe disease do when interacting/socializing with others?**	**Phenotype, Transplant status**	**Implications**
Our family is not very social, have a couple friends with younger kids- it's more on the other kids if they interact, my friends son includes him, asks about him, etc. most other kids don't pay him any attention. My son also loves books so when he and my friends son are together they will read, play on his tablet, things like that.	IKD, Transplanted early	Introverted, has a small group of freinds
She interacts socially but unable to physically join in- on par with other kids for socialization	IKD, Transplanted early	Able to interact socially, limited by physical abilities
He is very social, loves people, talking to people, making people smile and laugh, he's a jokester, has the best personality and just makes you laugh	IKD, Transplanted early	Loves to interact and socialize
He doesn't interact really, the older brother interacts but he doesn't give him anything back	IKD, Not transplanted/transplanted late	Minimal ability to interact and socialize with others
She is naive because she's sheltered but socially more mature than others, knows social engagement, appropriate with being out and about	LIKD, Not transplanted/transplanted late	Appropriate level of socialization
His brother is the one who can get him to laugh, tries to play with his brother, when my son is playing with another kid he will look at them back and forth, he is trying, never lost that	LIKD, Not transplanted/transplanted late	Tries to socialize but marked limitations

He definitely has more of an interest in babies and kids than adults, his two little cousins dote upon him and he watches them more than his grandparents, they're more interesting to him and make him smile a lot	LIKD, Transplanted early	Very socialbel, able to babysit little cousins
**Responses to: How has your sleep been impacted by caring for someone with Krabbe disease and what is it like?**	**Phenotype, Transplant status**	**Implications**
He still sleeps with me, we sleep pretty good and me and my husband take shifts. My son will sleep from 10-6 but he wakes up at 2,3,4 and he's ready to go for the day. Right now it's pretty good, just have to move him off me sometimes	IKD, Transplanted early	Spouses take turns caring for child, they and child sleep well
For years now I have not had a full night's sleep, I cat nap here and there. I never fall into a deep sleep, I will be sleeping but I hear everyone talking, last couple years I have some health problems, blood pressure is high, problems with weight, thyroid issues, keep losing eyesight, and my anxiety- really bad anxiety	IKD, Transplanted early	Markedly impaired sleep contibuting to worsening health
It's absolutely impacted, i am up every couple of hours checking on him, he wears a bipap and if it needs adjusted or has a leak, my husband and I take shifts - can't tell you the last time I slept 7 hours consecutively. Even if my husband takes over my body is still programmed to wake up every couple of hours. We have attempted to get nursing services - he qualifies for 16 hours a day of nursing staff, not enough applicants in the area, supports are available but no one to fill them.	IKD, Not transplanted/transplanted late	Markedly impaired sleep
All of it, there were nights she woke us up multiple times in the night, she always slept in our bedroom, had her own bed, at a year old we got a twin mattress so we could crawl in with her, it helped a little but before she was properly medicated so first 6-9 months we slept upright in bed holding her, once meds were in order she would sleep in her own bed, she would wake us up from 2-5 times a night needing to be suctioned, would giver her last feed at 11. [We] maybe got 5 hours of sleep on average.	IKD Not transplanted/transplanted late	Markedly impaired sleep due to heightend vigilance and frequent healthcare needs
It varies day to day- some days I sleep pretty well and some days I am up a lot, never sleep completely through the night because of my daughter, I wake up to change and reposition her, usually up around 4:30-6 maybe 5-6 hours of sleep	LIKD, Transplanted early	Impaired sleep, varies night-to-night
Falling asleep is most difficult, I have alarms go off 3 times in the night for water flush, meds, and to start a pump feed, falling asleep is hard because it might take me a bit to go back but falling asleep in general is difficult, about 4.5 hours of sleep	LIKD, Not transplanted/transplanted late	Markedly impaired sleep due to heightend vigilance and frequent healthcare needs
I am kind of on a routine but there are days I will not sleep, there are some days I can sleep okay, not sleeping can be either mental or being up with them, on a typical night I get 5-6 hours sometimes, it depends, throughout the night I get up and reposition and change her, may get up 2-3 times and that third time I'm up for the day	LIKD, Not transplanted/transplanted late	Impaired sleep, varies night-to-night

Appendix 1 contains selected caregiver remarks that illustrate different struggles involved in caring for a child with KD. Responses related to four categories were chosen. The four categories were as follows: 1) Missed educational/occupational opportunities due to caregiving for a child diagnosed with KD; 2) Impact of caring for child with KD on spousal relationship; 3) Impact on caregiver sleep due to having a child with KD; and 4) Ability for affected child to interact with others. The left column has the original caregiver quote. The middle column shows the ascribed cohort. The right column summarizes the implications conveyed by the quote.
